# Male Role Norms, Knowledge, Attitudes, and Perceptions of Colorectal Cancer Screening among Young Adult African American Men

**DOI:** 10.3389/fpubh.2014.00252

**Published:** 2014-11-27

**Authors:** Charles R. Rogers, Patricia Goodson

**Affiliations:** ^1^Program in Health Disparities Research, Family Medicine and Community Health, University of Minnesota Medical School, Minneapolis, MN, USA; ^2^Health and Kinesiology, Texas A&M University, College Station, TX, USA

**Keywords:** African American, cancer prevention, colon cancer, health disparities, masculinity, men

## Abstract

Racial disparities in health among African American men (AAM) in the United States are extensive. In contrast to their White counterparts, AAM have more illnesses and die younger. AAM have colorectal cancer (CRC) incidence and mortality rates 25% and 50% higher, respectively, than White men. Due to CRC’s younger age at presentation and high incidence among AAM, CRC screening (CRCS) is warranted at the age of 45 rather than 50, but little is known about younger AAM’s views of CRCS. Employing survey design, the purpose of the study was to describe the male role norms (MRN), knowledge, attitudes, perceived subjective norms, and perceived barriers associated with screening for CRC among a non-random sample of 157 young adult AAM (ages 19–45). Sixty-seven percent of the study sample received a passing knowledge score (85% or better), yet no significant differences were found among the three educational levels (i.e., low, medium, high). More negative attitudes toward CRCS correlated with the participants’ strong perceptions of barriers, but no extremely negative or positive MRN and perceived subjective norms were found. The factors significantly associated with attitudes were family history of cancer (unsure), work status, and perceived barriers. Findings from this study provide a solid basis for developing structured health education interventions that address the salient factors shaping young adult AAM’s view of CRC and early detection screening behaviors.

## Introduction

African American men (AAM) have more illnesses, die younger, and have less access to quality healthcare than White men in the United States (1–3). Compared to White men, AAM have incidence and mortality rates 25% and 50% higher, respectively, from colorectal cancer (CRC) (4). Johnson and colleagues reported that their sample of AAM and their physicians demonstrated lower levels of patient-centered communication in comparison to a sample of White men (5). Explicitly, research suggests AAM seldom see healthcare providers who are genuinely interested in their health concerns (6).

Since routine screening detects CRC at earlier, more treatable stages, the American Cancer Society, Rex and colleagues with the American College of Gastroenterology, and the U.S. Preventive Services Task Force recommend routine screening at age 50 for all men at average-risk using a combination of the following: yearly fecal occult blood test (FOBT), flexible sigmoidoscopy every 5 years, or colonoscopy every 10 years (7–9). Despite evidence that these three recommended early detection screening (EDS) practices can reduce CRC mortality, screening rates remain low among African Americans (10). Most men over age 50 have not undergone screening, and disparities in screening between AAM and their White counterparts persist (11).

Given the lower rates of CRC screening (CRCS) among AAM, for many providers the initiation of CRCS is warranted, in fact, at the age of 45 years rather than 50 years (8, 12). If true that earlier-than-50 screening is necessary, it may be beneficial to begin educating AAM about this preventable disease and the three EDS practices *before* age 50, given the documented disparities in new diagnoses and younger age at presentation of CRC among African Americans (8, 13). Unfortunately, CRCS uptake is also relatively low among AAM, a fact this is understudied and poorly explicated (14–17). Much of the extant research on CRC disparities impacting AAM has focused on modifiable lifestyle factors such as preventive screening, sedentary lifestyles, and low-fiber and high-fat diets, while neglecting how masculinity and social contextual factors shape those behaviors (16, 18, 19). The purpose of this study, therefore, was to describe the male role norms (MRN), knowledge, attitudes, perceived subjective norms, and perceived barriers associated with screening for CRC among younger-than-50 adult AAM (ages 19–45, specifically) employing survey research methods.

### Theoretical framework

**Figure 1:**
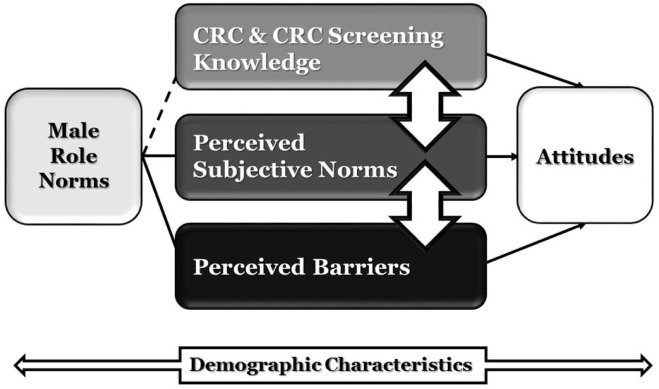
**Conceptual model of factors shaping attitudes toward CRC and CRCS among young adult African American men**.

This study was guided by a conceptual framework that integrated select concepts and constructs of the theory of planned behavior (TPB) (20) and perceptions of specific cultural norms related to male roles (21–25). Figure 1 postulates four factors that shape/affect a young adult African American male’s attitudes toward CRC and its prevention: MRN, knowledge, perceived subjective norms, and perceived barriers. The attitudinal factor refers to an African American male’s favorable or unfavorable beliefs and values associated with CRC and CRCS (15, 26, 27). Knowledge is the familiarity, awareness, or understanding of CRC and of the three recommended EDS practices (i.e., FOBT, Sigmoidoscopy, and Colonoscopy). The perceived subjective norms component deals with an African American male’s perception that important members of his support network value screening for CRC. Perceived barriers, originally added to the TPB by Ajzen as perceived behavioral control, accounts for those obstacles that stand in the way of a positive attitude or a specific behavior (28).

Male role norms are beliefs regarding rules, expectations, or social norms that dictate what an African American man considers an acceptable masculine attitude and behavior regarding CRCS, within a particular cultural and historical context (21, 25, 29). Masculinity, or prevalent MRN, has been identified as potentially dangerous to men’s health, as it plays a critical – but, oftentimes negative – role in healthcare use, mortality, and health behaviors of AAM in the U.S. (30–33). In this model, MRN represent how much men agree or disagree with an array of dominant cultural norms of masculinity (25, 34, 35). Ajzen and Fishbein grounded the TPB on the premise that behavior is a function of intention, attitudes, and specific perceptions (such as perceptions of subjective norms and behavioral control) (20). Yet, the TPB typically does not provide an explicit context for considering cultural values. Accordingly, MRN were included in the model and operationalized as the behaviors and attributes men should ideally embrace, based on sociocultural norms (36).

Since demographic variables contribute to health risk disparities among U.S. men and influence the kind of masculinity that men construct, demographic characteristics were included as control variables or covariates in the statistical analyses (30). These factors include age, marital status, sexual orientation, educational level, household income, work status, health insurance, and religious preference. Research suggests these factors influence both the perceptions of CRC as well as the behaviors of screening for CRCS (37).

## Materials and Methods

### Data source and study sample

From March to June 2013, a convenience and snowball sampling plan was used to recruit young adult AAM via a third-party on-line survey engine (PsychData). Inclusion criteria consisted of (a) young adult (ages 19–45), (b) men who self-described as African American, (c) resided in the U.S., and (d) were able to speak and understand the English language. A non-random sample of 207 young adult AAM who completed the on-line survey were assessed for eligibility and 157 met full inclusion criteria after missing data issues were resolved.

Participants were recruited nationally through various existing social networks such as list-serves, on-line networks (e.g., Facebook, Twitter), predominantly African American-serving barbershops, National Pan-Hellenic Council fraternities, African American male-dominant organizations, and predominately African American mega-churches. Study protocol was approved by the Institutional Review Board (IRB) at Texas A&M University.

### Data collection

Data collection was performed through a survey questionnaire administered on-line with the assistance of PsychData (an on-line survey tool explicitly designed to meet and surpass IRB standards for the protection of research participants along with industry standards for Internet security) (38).

The survey was located at a domain titled “ChangeThaGame.com”. When participants visited this site, they were informed that the playing field is not even as it relates to deaths from CRC for AAM. They learned their participation was requested to begin addressing this complex issue and assure a win in their (i.e., the young adult African American male participants) favor. The webpage contained an information sheet, which stated that participation was voluntary and would last approximately 30 min. After reading the information sheet about the study posted on the website, participants were asked to certify that they were an African American male between the ages of 19 and 45, and advised to select yes or no in regards to giving consent to participate in the study. By selecting yes, participants began the survey. Upon completing the survey, participants were given the choice to be entered into four random drawings to win one of four incentives in the form of electronic devices commonly wanted by men.

### Measures

Two previously developed instruments were employed in this study: (a) the *Male Role Norms Inventory-Short Form* (MRNI-SF) developed by Levant, Hall, and Rankin (39), and (b) a modified version of the *CRC Knowledge and Perceptions Survey for Older African Americans Survey* developed by Green and Kelly (40). Items from both instruments, employed with permission, assessed MRN, knowledge, and perceptions. The first author developed the items measuring all other constructs. Table 1 provides examples of how each of the constructs in the theoretical model was operationalized for this study.

**Table 1:** **Samples of items used in the survey of African American young adult men, to assess their male role norms, knowledge, attitudes, and perceptions of CRCS**.

Item number in survey	Question	Demographic characteristics	Construct: male role norms	Construct: CRC and screening knowledge	Construct: attitudes toward CRC screening	Construct: perceived subjective norms	Construct: perceived barriers
1	What race do you self-identify as?	**X**					
20	Homosexuals should never marry.		**X**				
41	Colorectal cancer is a cancer of the colon or rectum.			**X**			
62	The thought of getting colorectal cancer scares me.				**X**		
14	Are you currently active/participate in any type of male-dominant social group (e.g., AAU basketball, fraternity, bowling league, Bible study group)?					**X**	
13	Do you have one doctor who you continually connect with (i.e., primary care/family physician)?						**X**

The on-line instrument was pilot-tested with a small convenience sample of nine undergraduate and graduate students (aged 22–48). With this sample, “cognitive interviewing,” a process that elicits input from participants as they respond to the survey in real time, was conducted. This method provided further assurance that the participants and researchers had a shared understanding about the meaning of the items on the survey, hence, enhancing its internal validity (41). Specifically, the interviewer asked participants: (a) how they understood each question and respective response options; (b) whether the question was likely to elicit an honest response, in the field; (c) whether specific wording of questions communicated adequately or should be changed/adapted; and (d) the user-friendliness of the on-line setup for the questionnaire, among other process-oriented questions. The pilot-testing also allowed assessing participants’ comfort-level with using PsychData, and whether any built-in skip patterns functioned as planned.

#### Demographic characteristics

Participants’ demographic characteristics assessed in Part I of the survey included: race, gender, age, current residence, marital status, sexual orientation, highest level of educational attainment, formal association with any health related field (e.g., pursuing or holding a degree in health education, public health, nursing, allied health), household income, work status, health insurance status, religious preference, church attendance, family history of cancer, family history of CRC, and history of CRC (self). This section also included two questions inquiring how participants learned about the study and if they had a doctor they saw on a regular basis.

#### Male role norms

Part II of the survey consisted of 21 items, with response options on a 7-point Likert scale (1 = *strongly disagree*, 7 = *strongly agree*), from the MRNI-SF (39). The form comprises the subscales: avoidance of CRC and early detection screening knowledge, beliefs, and values. Femininity, negativity toward sexual minorities, self-reliance through mechanical skills, toughness, dominance, importance of sex, and restrictive emotionality. Higher scores “indicate higher levels of endorsement of traditional masculinity ideology” (p. 230) (39).

#### CRC and early detection screening knowledge, beliefs, and values

Part III of the survey included 46 items divided into two sections stemming from a modified version of the *CRC Knowledge, Perceptions, and Screening Surve*y originally developed by Green and Kelly (40): knowledge about CRC and EDS, and beliefs and values about CRC and EDS. Section 1, the *Knowledge about CRC and EDS* scale, consisted of 21 true/false items on CRC warning signs and symptoms, incidence and mortality, truths and myths, participation in screening, and screening modalities. After exploratory factor analysis, eight items were removed to improve the reliability and validity of the knowledge scores. Each item was assigned 1 point if correct for a total of 13 possible points or 100%. Participants had to answer 11 out of 13 questions correctly (85%) to receive a “passing” score. Section 1 was initially adapted by Green and Kelly from the 18-item Breast Cancer Knowledge test by McCance, Mooney, Smith, and Field (40, 42).

Section 2, the *Beliefs and Values about CRC and EDS* scale, consisted of 54 items on a 5-point Likert scale (1 = strongly disagree, 5 = strongly agree), measuring CRC severity, screening benefits, screening barriers, and perceived subjective norms. Later, the scale was sub-divided into three subscales: the *Attitudes subscale*, the *Perceived Barriers subscale*, and the *Perceived Subjective Norms subscale*. The items in Section 2 were adapted by Green and Kelly from a scale developed by Champion and Scott (40, 43).

### Data analysis

Data from the survey were imported into SPSS from the survey engine, PsychData, and analyzed using version SPSS 20.0. Alongside descriptive statistics, multiple regression was employed as the primary analysis to explore the relationships among the proposed constructs. To test for potential moderating effects, data were divided into those with health insurance versus those without, and those with high school or less education, versus those with some college or above. Age was treated as a continuous variable.

Descriptive statistics (frequencies, percentages, means), bivariate analyses (Pearson’s *r*, Chi-Square), and multiple regression assessed the characteristics and relationships among the factors in this study, explored potential group differences, and tested moderator effects. An α of 0.05 was proposed as the critical value of probability. All variables were tested for normality and other relevant assumptions, and r not to have violated any of them (44).

### Missing data

Missing data were thoroughly reviewed to explore the “*mechanism of missingness* – that is, the hypothesized reason for why data are missing” (p. 109) (45). This revision revealed the data appeared to be missing not at random (MNAR). Due to (a) the difficulties that employing techniques to handle MNAR data can bring to interpretation (46); and (b) the potential for biasing results (47), participants with MNAR responses were removed from the sample. Supporting our decision not to impute scores or otherwise supply data for the missing values was the small amount of missing data in our sample (*n* = 2). Participants who did not respond to at least 94% of the survey (*n* = 11) also were removed from the final sample. Three participants did not give consent to participate and four gave consent yet did not fill out the survey, so the data for all seven were removed. Additionally, 17 participants who did not meet the 19- to 45-year-old, adult African American male inclusion criteria were removed from the final sample. Lastly, the data were carefully scrutinized to determine whether the same participants completed the survey more than once or whether several computers in a common computer lab were used (sharing the same IP address). For cases that were similar (*n* = 3), only one was retained for the final sample.

## Results

### Description of sample

A total of 207 surveys were assessed for eligibility and 157 met full inclusion criteria after missing data issues were resolved. Study participants had a mean age of 29.78 ± 5.87. Specifically, 47% were single, 103 were 25–35 years old (68%), and nearly half had a Master’s/advanced degree (45%). The median household annual income was $35,000–$49,000; the majority worked full-time (62%) and had health insurance (83%). Regarding the four regional divisions used by the U.S. Census Bureau, 77% of participants resided in the South, followed by the Midwest (12%), Northeast (7%), and Western regions of the country (4%).

Regarding cancer history, only 2% (*n* = 3) had been diagnosed with CRC. While 68% did not have a family history of CRC, 40% claimed a family history of *cancer*. In terms of having a primary care/family physician, 52% had a doctor whom they saw on a regular basis. In terms of participation/enrollment in the study, 48% learned about it because their friends/family member/someone told them about it, 24% via Facebook/Twitter, and 22% via email/common interest list-serves. Half of the sample (52%) was currently active/participating in some form of male-dominant social group (e.g., AAU basketball, fraternity, bowling league, Bible study group). Additional participant demographic characteristics are presented in Table 2.

**Table 2:** **Participant demographic characteristics**^a^.

Sample characteristics (*N* = 157)	*n*	%
Age
19–24	28	17.8
25–35	103	65.6
36–45	26	16.6
Current residence
Midwest	19	12.1
Northeast	11	7.0
South	121	77.1
West	6	3.8
Marital status
Single	73	46.5
Unmarried in a relationship	29	18.5
Married	45	28.7
Divorced	4	2.5
Separated	3	1.9
Widowed	1	0.6
Sexual orientation
Straight	140	89.2
Gay	12	7.6
I am struggling with my sexual orientation	4	2.5
Highest education level completed
High school diploma	7	4.5
Partial college (at least 1 year)	22	14.0
Two year college/associate degree	5	3.2
Bachelor’s degree	52	33.1
Master’s/advanced degree	71	45.2
Are you currently pursuing or already have a degree in health education, public health, community health, or any health related field (e.g., nursing, allied health)?
Yes	33	21.0
No	124	79.0
Household income per year
<$15,000	24	15.3
$15,000–$24,999	21	13.4
$25,000–$34,999	16	10.2
$35,000–$49,000	20	12.7
$50,000–$74,000	26	16.6
>$75,000	50	31.8
Do you currently work (please select all that apply)?
No	8	5.1
Yes, part-time	28	17.8
Yes, full-time	98	62.4
Student	23	14.6

*^a^Information that does not add up to *N* = 157 (100%) is a result of data that were not reported*.

### Data validity and reliability

Psychometric analyses were conducted to establish the data’s validity and reliability. Specifically, exploratory principal component factor analysis with Varimax rotation conducted with all the major scales and subscales revealed the need to eliminate eight items from the *Knowledge about CRC and EDS* scale, six items from the *Attitudes* subscale, and one item from the *Perceived Subjective Norms* subscale.

Cronbach’s alpha was calculated, to assess whether the scores on the 21 items that were summed to create the *male role norms* scale, the 21 items in the *Knowledge about CRC and EDS* scale, the 17 items in the *attitudes subscale*, the 10 items in the *perceived subjective norm subscale*, and the 4 items in the *perceived barriers subscale* were internally consistent. The lowest-scoring scales were the knowledge scale (α = 0.45) and the *perceived barriers subscale* (α = 0.71). All other scales had reliability coefficients close to, or above, 0.80 (role norms α = 0.90; attitudes α = 0.79; perceived subjective norms α = 0.87). The low score for *Knowledge about CRC and EDS* was not surprising for an index assessing a cognitive/recall-type variable, because knowledge of various *dimensions* of CRC and CRCS was measured. Aside from the knowledge about CRC and EDS scale, the strong reliability coefficients for all other scales suggest the variables/scales had acceptable-to-good levels of internal consistency or score reliability (48). Based on exploratory factor analysis findings (see below), eight items were deleted from the final *Knowledge about CRC and EDS scale*. The alpha for the re-defined scale improved (α = 0.54).

The alphas for the MRNI-SF were evaluated using the criteria developed by Ponterotto and Ruckdeschel which involves clustering scale lengths into three general ratings (e.g., moderate, good, and excellent) to consider “the adequacy of magnitudes for coefficient alpha in light of item count and sample size” (p. 1002) (49). For the MRNI-SF subscales and total scale, the alphas ranged from good (0.80–0.84) to excellent (0.85 and up), with the exceptions of the moderate (0.75–0.79) alphas observed for the subscales of self-reliance through mechanical skills (0.79), negativity toward sexual minorities (0.78), toughness (0.79), and dominance (0.79). The general traditional masculinity ideology factor (MRNI-SF total score) was excellent (0.90).

### Male role norms

Male role norms were measured by 21 items, with response options on a 7-point Likert-type scale (1 = *strongly disagree*, 7 = *strongly agree*). For descriptive analysis purposes, the level of agreement or disagreement was determined by combining those who responded strongly agree with those who responded agree, and similarly for those who disagreed and strongly disagreed. The statement with the highest percentage of participants agreeing/strongly agreeing was *when the going gets tough, men should get going* (50%) followed by 46% who agreed/strongly agreed *men should have home improvement skills*. Conversely, the highest percentage of disagreement/strong disagreement was for the statement *a man should never admit when others hurt his feelings* (79%) followed by 74% who disagreed/strongly disagreed *the President of the U.S. should always be a man*.

The mean scores for the sample (*n* = 143) on the MRN section of the survey ranged from 1.97 to 5.10 for each question. The sample had a total mean score of 3.33 (SD = 1.00). This indicates the men, on average, slightly *disagreed* with endorsing a traditional masculinity ideology. Forty-one percent of the participants scored below the group’s mean score meaning they disagreed/strongly disagreed with male role norm items.

### CRC and screening knowledge

The statement with the highest percentage of participants responding correctly was *CRC is a disease that affects only older, white men* (99% – false statement) followed by *CRC is a cancer of the colon or rectum* (98% – true statement), *bleeding from the rectum*, *blood in your stool*, or *blood in the toilet after a bowel movement may be symptoms of CRC* (98% – true statement), *the risk of developing CRC is greater as a person gets older* (94% – true statement), *CRC is the leading cause of cancer death in the* U.S. (89% – false statement), *most colorectal cancers begin as a growth in the colon or rectum* (89% – true), *CRC is the third most common cancer in African Americans* (83% – true statement), and *AAM should begin screening for CRC at age 45* (76% – true statement). Conversely, the highest percentage of incorrect responses was for the statement *men and women should begin screening for CRC soon after turning 50 years of age* (39% – true statement) followed by *a sigmoidoscopy is an appropriate test to screen for CRC* (33% – true statement).

Although three different screening tests are recommended for CRC, participants differed in agreeing that a Colonoscopy (89%), FOBT (85%), and Sigmoidoscopy (67%) are appropriate for testing. Ninety-eight percent of the participants responded correctly to the true statement *bleeding from the rectum*, *blood in your stool*, or *blood in the toilet after a bowel movement may be symptoms of CRC*.

The scores for the total sample on the Knowledge about CRC and EDS scale ranged from 6 to 13 with a mean score of 11.02 (SD = 1.65). In order to receive a passing score, participants were expected to answer 11 out of 13 questions correctly (85%). Sixty-seven percent of the study sample received a passing score (*n* = 105), of which 22% received a perfect score of 100% (*n* = 34).

Before examining the relationship between education and CRC-and-screening-knowledge, the seven education categories in the survey instrument were combined into three levels: *low* (partial high school, GED or equivalent, and high school diploma), *medium* (partial college and 2 years college/associate degree), and *high* (Bachelor’s degree and Master’s/Advanced degree). A one-way ANOVA identified no significant differences in the mean Knowledge about CRC and EDS scores among the low (*M* = 11.71, SD = 1.11), medium (*M* = 10.85, SD = 1.49), and high (*M* = 11.02, SD = 1.71) education groups, *F* (2, 154) = 0.750, *p* = 0.474.

### Attitudes toward CRC and CRCS

The attitudes toward CRC and CRCS were initially measured by the responses to seven items covering CRC severity, five items addressing screening benefits, and five items covering screening barriers from the Beliefs and Values about CRC and EDS scale. After exploratory factor analysis, five items (i.e., four items addressing screening benefits and one item addressing screening barriers) were removed to improve the reliability and validity of the final *attitudes subscale*. All items were on a 5-point Likert-type scale (1 = strongly disagree, 5 = strongly agree). For descriptive analysis purposes, the level of agreement or disagreement was determined by combining those who responded strongly agree with those who responded agree, and similarly for those who disagreed and strongly disagreed. Regarding CRC severity, 71% agreed/strongly agreed *the thought of getting colorectal cancer scares me*; and 59% agreed/strongly agreed *if I got colorectal cancer*, *my whole life would change*. Conversely, 74% disagreed/strongly disagreed with the statement *if I had colorectal cancer*, *my career/life would be over*. Furthermore, a large segment believed screening can decrease mortality: 83% agreed/strongly agreed that *having colorectal cancer screening will decrease my chances of dying from colorectal cancer*.

Of the four items that measured perceptions of screening barriers, the highest percentage of *disagreement* were responses to *colorectal cancer screening is embarrassing to me* (62%). Forty-one percent (41%) admitted being *afraid to find out there is something wrong when I have colorectal cancer screening*, and 21% were *afraid to have colorectal cancer screening because I do not understand what will be done*.

The mean score for the total sample (*n* = 149) on the Attitudes toward CRCS section of the survey ranged from 1 to 4.75 for each question with a total mean score of 2.91 (SD = 0.617) for the composite scale (range of 1–5 for possible scores). Fifty-one percent of the participants scored above the group’s mean score, indicating the sample was equally split in terms of positive and negative attitudes.

Many private insurance plans as well as Medicare Part B (medical insurance) cover several types of EDS tests for CRC (50, 51). Thus, the researchers wanted to explore whether attitudes toward EDS test for CRC varied according to this important factor (having health insurance). A one-way ANOVA was run and no statistically significant differences in the mean scores for attitude toward CRCS among those with and without health insurance were found, *F* (1, 147) = 0.612, *p* = 0.435.

### Perceived subjective norms

Perceived subjective norms were measured by 10 items from the Beliefs and Values about CRC and EDS scale. These items were on a 5-point Likert-type scale (1 = strongly agree, 5 = strongly agree). For descriptive analysis purposes, strongly agree and agree responses were combined, as were disagreed and strongly disagreed. The highest percentages of agreement/strong agreement with the items measuring perceived subjective norms were responses to *it is important for me to do what important people in my life think is appropriate* (61%) and *the important people in my life believe colorectal cancer screening can help prevent colorectal cancer* (56%). The item, *it is important for me to comply with what my* “*significant other*” *believes in*, exhibited the lowest proportion of agreement/strong agreement (54%).

The mean scores for the sample (*n* = 149) on the perceived subjective norms subscale ranged from 3.16 to 3.64 for each question with a total mean score of 3.45 (SD = 0.637) for the composite scale – meaning that, overall, the men in our sample were rather ambivalent regarding subjective norms. Specifically, 49% of the participants scored below the group’s mean score, indicating that, similar to their attitudes, the sample was split between weaker and stronger perceptions of subjective norms.

### Perceived barriers

Perceived barriers were measured by the responses to four items on screening barriers from the Beliefs and Values about CRC and EDS scale. The four items on screening barriers were on a 5-point Likert-type scale (1 = strongly disagree, 5 = strongly agree). As the researchers did for the previous scales, strongly agree/agree responses were combined, as well as strongly disagreed/disagreed. Among items that measured perceived subjective norms, more than 50% of the sample disagreed with 3 of the statements. The highest percentages of disagreement/strong disagreement were responses to *having colorectal screening could take too much time* (69%) and *having colorectal screening would expose me to too much radiation* (54%).

The mean scores for the sample (*n* = 153) on the perceived barriers subscale ranged from 2.17 to 2.74 for each question with a total mean score of 2.47 (SD = 0.719) for the composite variable. Fifty-five percent of the participants scored above the group’s mean score, indicating the majority of participants had a strong awareness of potential barriers to CRCS.

### Factors shaping attitudes toward CRC and CRCS

To examine the relationships among MRN, knowledge, perceived subjective norms, perceived barriers, and attitudes, while controlling for various demographic characteristics of the sample, a series of multiple regression models were run with attitudes as the predicted variable. Table 3 presents these models, and allows for comparisons among predictors for each model. Multicollinearity did not represent a problem for any of the models tested as none of the variance inflation factors (VIF) associated with the predictor variable was greater than 10. The assumption of homoscedasticity and linearity were not violated for any of the models tested as the scatterplot of the regression standardized residuals and the regression standardized predicted values were evenly scattered around zero, while the normal P–P plot of the regression standardized residual for the dependent variable was fairly linear.

**Table 3:** **Standardized beta coefficients for predictors of *attitudes toward colorectal cancer screening*, according to regression models**.

Predictors	Model 1A Adj. *R*^2^ = 0.143 β	Model 1B* Adj. *R*^2^ = 0.068 β	Model 1C* Adj. *R*^2^ = 0.223 β	Model 2 Adj. *R*^2^ = 0.247 β	Model 3 Adj. *R*^2^ = 0.272 β	Model 4 Adj. *R*^2^ = 0.236 β	Model 5 Adj. *R*^2^ = 0.276 β	Model 6** Adj. *R*^2^ = 0.417 β
Demographic characteristics
Age	−1.27		− 0.161	− 0.177	− 0.189	− 0.187	− 0.097	− 0.063
NE region	0.103		0.105	0.087	0.068	0.1	0.073	0.088
MW region	0.042		0.05	0.069	0.054	0.032	0.063	− 0.011
W region	0.089		0.084	0.07	0.066	0.075	0.05	0.098
Gay orientation	−0.173		− 0.157	− 0.145	− 0.122	− 0.15	− 0.143	− 0.151
Struggling orientation	−0.235		− 0.114	− 0.095	− 0.09	− 0.122	− 0.069	− 0.081
Married	−0.065		− 0.012	− 0.016	0.012	0	− 0.045	− 0.069
Separated	−0.033		0.116	0.127	0.103	0.086	0.104	0.84
Med. education	0.123		− 0.112	− 0.129	− 0.042	− 0.051	− 0.068	− 0.129
Adv. education	−0.065		− 0.028	− 0.084	− 0.048	− 0.014	− 0.042	− 0.129
Low income	−0.033		− 0.04	− 0.079	− 0.06	− 0.021	− 0.092	− 0.198
Middle income	0.123		0.092	0.026	0.026	0.081	0.033	− 0.034
Work status	−0.172		− 0.191	−0.235*	−0.238	− 0.201	− 0.165	−0.245*
Health insurance	0.165		0.166	0.104	0.12	0.192	0.051	0.138
Practicing Christian	−0.199		− 0.155	− 0.029	− 0.031	− 0.158	− 0.094	− 0.019
Practicing other	−0.066		− 0.018	0.005	− 0.019	− 0.025	− 0.037	− 0.075
Nominal other	−0.111		− 0.116	− 0.067	− 0.052	− 0.1	− 0.086	− 0.021
Family history
Sure of family history of cancer		0.152	0.128	0.115	0.096	0.105	0.058	0.167
Unsure of family history of cancer		0.286**	0.351**	0.281*	0.281*	0.342**	0.201	0.204
Sure of family history of CRC		− 0.453	− 0.012	− 0.04	− 0.057	− 0.005	− 0.069	− 0.031
Unsure of family history CRC		− 0.194	− 0.465	0.012	0.029	− 0.029	0.059	0.005
Male role norms
Negative femininity				− 0.012	− 0.022		0.015	0.004
Male dominance				− 0.213	− 0.228		− 0.252	− 0.188
Self-reliance through mechanical skills				0.111	0.125		0.073	0.028
Restrictive emotion				0.159	0.185		0.165	0.07
Importance of sex				0.165	0.152		0.151	0.139
Toughness				− 0.149	− 0.109		− 0.147	− 0.154
Knowledge
Knowledge 1					0.064	0.019		
Knowledge 2					− 0.003	0.001		
Subjective norms							0.213	
Perceived barriers								0.505***

*p < 0.05; **p < 0.01; ***p < 0.001.

*Only statistically significant predictors are shown*.

#### Model 1A

A multiple linear regression was calculated predicting participants’ attitudes based on their demographic characteristics. In our study, demographic characteristics included the following: age, current state of residence according to the four U.S. Census Bureau-designated areas (i.e., Northeast, Midwest, South, and West), marital status, sexual orientation, education level, Health insurance, household income per year, religiosity, and work status.

The regression equation was not significant [*F*(17, 109) = 1.068, *p* = 0.394] with an adjusted *R*^2^ of 0.143. None of the demographic variables examined predicted participants’ attitudes toward CRCS (see Table 3).

#### Model 1B

This model examined participants’ attitudes in relationship to their family history of cancer, and no other covariates. This time, the set of predictors, as a whole, significantly predicted attitudes, *F*(4, 144) = 2.633, *p* = 0.037, but did not explain much of the variance (adjusted *R*^2^ = 0.068). In the analysis, family history of cancer consisted of four variables coded as sure of family history of cancer (participants have a family history of cancer), unsure of family history of cancer (participants are unsure of their family history of cancer), sure of family history of CRC (participants have a family history of CRC), and unsure of family history of CRC (participants are unsure of their family history of CRC). No family history of cancer and CRC were the reference groups.

Family history of cancer was significantly associated with attitudes toward screening for CRC, in this sample. Compared to those that do not have a family history of cancer, those that were *unsure* of their family history of cancer had a significantly better attitude toward screening for CRC, keeping all other covariates constant (β = 0.286, *p* = 0.005; see Table 3).

#### Model 1C

In this model, the researchers controlled for participants’ demographic characteristics, when examining the relationship between family history of cancer and attitudes. With the covariates, the regression equation, overall, was not significant [*F*(21, 105) = 1.437, *p* = 0.118] with an adjusted *R*^2^ of 0.223. Yet, family history of cancer maintained its significant relationship with attitudes. Compared to those that do not have a family history of cancer, those that were *unsure* of their family history of cancer had a significantly better attitude toward screening for CRC, after controlling for covariates (β = 0.351, *p* = 0.003; see Table 3).

#### Model 2

In this model, the relationship of MRN with attitudes was examined, adding the six factors in the *MRN scale* as separate predictors in the equation, along with demographic characteristics and family history of cancer. The regression equation was not significant [*F*(27, 90) = 1.092, *p* = 0.367] with an adjusted *R*^2^ of 0.247. Compared to those that do not have a family history of cancer, those that were *unsure* of their family history of cancer had a significantly better attitude toward screening for CRC, keeping all other covariates constant (β = 0.281, *p* = 0.030). Compared to those that work, those that do not work had a significantly *worse* attitude toward screening for CRC, keeping all other covariates constant (β = −0.235, *p* = 0.037; see Table 3)^1^. Table 3 shows standardized beta coefficients for predictors of attitudes toward colorectal cancer screening, according to regression models.

#### Model 3

This model assessed whether attitudes toward CRC and CRCS were related to demographic characteristics, family history of cancer, *male role norms* broken down as six factors, and knowledge. The regression equation was not significant [*F*(29, 83) = 1.068, *p* = 0.396] with an adjusted *R*^2^ of 0.272. Compared to those that do not have a family history of cancer, those that were *unsure* of their family history of cancer had a significantly better attitude toward screening for CRC, keeping all other covariates constant (β = 0.281, *p* = 0.033; see Table 3).

#### Model 4

In this model, the variable MRN was removed and the equation estimated participants’ attitudes based on their SES, family history of cancer, and knowledge. Once again, the regression equation was not significant [*F*(23, 97) = 1.300, *p* = 0.188] with an adjusted *R*^2^ of 0.236. Yet, family history of cancer maintained its role as significant predictor of attitudes toward screening for colorectal cancer. Compared to those that do not have a family history of cancer, those that were *unsure* of their family history of cancer had a significantly better attitude toward screening for CRC, keeping all other covariates constant (β = 0.342, *p* = 0.005; see Table 3).

#### Model 5

In this model, the role of perceived subjective norms was examined, added to the prediction equation, along with demographic characteristics, family history of cancer, and MRN broken down as 6 factors. The regression equation was not significant [*F*(28, 85) = 1.156, *p* = 0.300] with an adjusted *R*^2^ of 0.276. In this model, family history of cancer lost its significant association with attitudes, and none of the other variables predict participants’ attitudes toward CRCS (see Table 3)^2^.

#### Model 6

This model examined the role that *perceived barriers* might play in participants’ attitudes. A multiple linear regression was calculated to predict participants’ attitudes based on their demographic characteristics, family history of cancer, MRN broken down as six factors, and perceived barriers. This time, the set of predictors, as whole, significantly predicted attitudes, *F*(28, 89) = 2.278, *p* = 0.002, with a modest effect size (adjusted *R*^2^ = 0.417). Specifically, participants’ perceptions of barriers toward CRCS and their work status significantly predicted attitudes. Stronger perceptions of barriers (i.e., agreeing/strongly agreeing there are several barriers to screening) was significantly associated with more negative attitudes toward screening (β = 0.505, *p* = 0.000). To recall, negative attitudes were represented by higher scores on the attitudes scale. Compared to those that worked, those that did not work had a significantly *worse* attitude toward screening for CRC, keeping all other covariates constant (β = −0.245, *p* = 0.014; see Table 3).

## Discussion

By employing survey research methodology, this study provided valuable information on the MRN, knowledge, attitudes, perceived subjective norms, and perceived barriers associated with screening for CRC among young adult AAM (ages 19–45, specifically). Ultimately, *family history of cancer*, *work status*, and *perceived barriers* were the critical factors associated with attitudes in all of the models/analyses. Of these factors, perceived barriers are the one most amenable to change through public health education efforts.

### Male role norms

In designing this study, the researchers had anticipated that the participants’ beliefs regarding norms of masculinity would be critical for shaping their attitudes toward CRC and CRCS. Contrary to expectations, however, scores on the MRN scale were not associated with scores for attitudes toward CRC and CRCS, when controlling for several covariates. This finding suggests that MRN may influence CRCS through different mechanisms or pathways, rather than through shaping attitudes toward CRC/CRCS, directly, at least in our study’s sample.

The social constructions of masculinity and their influence on men’s well-being are of the utmost importance, yet research that studies how MRN shape young adult AAM’s attitudes toward CRCS is conspicuously absent. Courtenay explored how such factors as social context, educational level, economic status, sexual orientation, and ethnicity influence men’s construal of masculinity and contribute to differential health risks among men in the U.S. (30). He argued, “some men do defy social prescriptions of masculinity and adopt health behaviors, such as getting annual physicals and eating healthy foods. But although these men are constructing a form of masculinity, it is not among the dominant forms adopted by most men” (p. 1397) (30). Griffith and colleagues strongly agree, and add that masculinity plays a critical role in the health of AAM specifically (52). Future research associated with masculinity ideologies should examine whether MRN directly affect intention to screen for CRC and screening behaviors, and/or whether there are other theoretically plausible mechanisms of influence.

Absence of a relationship between MRN and attitudes in our sample could be explained by measurement error, also, but tests of the validity and reliability of the MRN scale indicated the data were of adequate quality. Yet, it is important to note that, along with this intriguing finding, the problems associated with our missing data may suggest the need to assess the MRN scale, more carefully. While the scale has been recently tested by other researchers (39), in this study the researchers determined that a number of participants chose to withdraw after reaching the MRN questions associated with *Negativity toward Sexual Minorities*. This reaction can be telling, and deserves more attention in future studies, as it could lead to biased samples and biased measures.

### CRC and screening knowledge

Our participants’ average knowledge score was better than the mean knowledge scores for AAM (>50 years of age) sampled in the descriptive correlational study conducted by Green and Kelly, indicating that younger men in our study might be more knowledgeable about CRC (40). While no age differences related to knowledge among subgroups in our sample were identified, other studies have reported variations. For instance, in a study that examined CRCS knowledge and potential covariates (e.g., health care, cancer information seeking) among over 3,000 adults (>45 years of age) from the 2003 Health Information National Trends Survey (HINTS 2003), those who were ages 45–49 or over 70, were less likely to have adequate screening knowledge (53). This difference by age not only calls attention to the significant increase in CRCS knowledge at age 50 but also may indicate that providers are recommending CRCS at this age, exclusively.

Despite intensive promotion of screening after 50, our younger participants may be progressively developing a perception of risk for CRC, and may, therefore, be increasingly interested in learning about the illness and its prevention, much like their older counterparts. The fact that 76% of participants in our study answered correctly the knowledge item, *AAM should begin screening for colorectal cancer at age 45* (true statement), may suggest they might be hearing messages regarding earlier screenings.

Even though the knowledge items in this study performed adequately – the original Cronbach’s alpha was 0.45, and after removal of eight items, improved to 0.54 – better measures of knowledge are still needed to develop interventions addressing knowledge as a factor in CRCS (13, 54). Nonetheless, as indicated in this study, prevention efforts focusing solely on knowledge might be less-than-useful, given the absence of a direct relationship between knowledge and attitudes toward CRC and CRCS.

### Attitudes toward CRC and CRCS

The researchers initially expected that participants would espouse negative attitudes toward screening for CRC, and that these would be shaped by their perceptions of MRN. Although the researchers did find the sample held more negative attitudes toward CRC and CRCS, rather than positive, MRN had no association with attitudes.

Instead, the authors observed an interesting interplay between perceived subjective norms and family history: subjective norms overrode family history (i.e., family history of cancer lost its significant association with attitudes in the presence of subjective norms), yet still did not predict attitudes. The authors believe this phenomenon provides clues for future program development: something seems to be “going on” related to both family history and perceived subjective norms – something our sample was not able to capture entirely, but other studies might explore. For program planning purposes, subjective norms and family history variables might offer better “starting-points” than other factors commonly targeted in prevention programs (e.g., MRN and knowledge) for shaping young AAM’s attitudes toward CRC and CRCS. In this study, the statistical models did not provide a clear-cut picture but their behavior suggests the potential salience of these variables. Future research would do well to explore these relationships, even further.

Findings from our study supported the internal and external barriers commonly reported in other studies (where the minimum age to participate was 50), such as pain, fear of cancer diagnosis, embarrassment, cost of screening tests, cost of treatment if diagnosed with CRC, absence of recommendation by primary physician, and/or health care provider (55, 56). For instance, James and colleagues investigated perceived barriers and benefits to CRCS among 850 African American adults participating in a church-based health promotion program in rural northern North Carolina (55). Among this over-50 sample, participants “with a stronger perception of barriers were less likely to report a recent FOBT, but higher perceived benefits did not significantly affect FOBT rates. A similar pattern for perceived barriers emerged with sigmoidoscopy, in which higher scores on perceived barriers were associated with lower rates of recent sigmoidoscopy” (p. 532) (55).

More than half of our participants agreed/strongly agreed that *the thought of getting CRC scares me* and *if I got CRC*, *my whole life would change*. Forty-one percent admitted being *afraid to find out there is something wrong when I have CRCS*, and 21% were *afraid to have CRCS because I do not understand what will be done*.

Fear and anxiety have been well documented in the literature as a barrier to CRCS among African Americans (57, 58). For instance, fear of medical procedures and receiving a morbid diagnosis were cited as significant barriers to seeking medical care among the AAM in studies by Ravenell et al. (59) and Sly et al. (60). Similarly, fear, dislike, and apprehension accounted for nearly 50% of the reasons given by 179 study participants in Virginia, for not seeking CRCS (61). While this finding was not unexpected, Good and colleagues argued how these feelings may have a historic background tied to the medical mistrust among African Americans that has been warranted by the legacy of previous medical research abuses, such as the Tuskegee Syphilis Study (61). The deception associated with the Tuskegee study in which 400 AAM were denied treatment for syphilis, as well as concerns about being treated as a “guinea pig,” frequently emerge in studies of African Americans’ attitudes toward any form of medical research (62, 63). Thus, research that strives to diminish this underlying issue of mistrust and fear must be addressed in order to improve the attitudes of young adult AAM toward future research associated with CRCS.

## Conclusion

That a couple of contextual variables emerged as strong, independent predictors of attitudes in most of the models – family history of cancer and work status – suggests to health educators the need to consider CRC and CRCS attitudes from an ecological perspective, where contextual variables such as employment and uncertainty about family history of illnesses play, perhaps, a more salient role than individuals’ knowledge and attitudes.

Whether work status is a factor that can be changed through health promotion efforts, however, is debatable. Simons-Morton, McLeory, and Wendel, for instance, argue:

“…community change and building community capacity to address health problems are major objectives of community-based interventions (.). In addition to incorporating community perspectives, using community resources, and strengthening community capacity, we [health promoters] may also work with and in communities to target the social conditions – unemployment, poverty, housing, law enforcement, gangs, education, the built environment – that produce and sustain many of the problems with which we are concerned, including violence, school dropouts, adolescent pregnancy, drug and alcohol use, physical inactivity, and obesity” (p. 57) (64).

Regardless of their ability to affect the work status of a population, health educators certainly *can* strive to promote the use of family health history tools (65, 66) and assure that those men who are “unsure” of their family history of cancer become more informed of steps to prevent CRC and other chronic diseases.

In terms of perceived barriers (e.g., fear, embarrassment, poor patient-provider communication), health educators, alone, cannot eliminate these barriers, yet they can become team players in partnerships among community-based organizations, public health professionals, transdisciplinary research teams, and policymakers. Health educators can – and should – contribute significantly to systemic changes that reverse the saddening reality of AAM, whose “health […] is the worst of any demographic group in the United States” (p. 331) (67).

### Limitations

Despite the contributions this study makes, it suffered from important limitations. Selection bias may have been present as only those who were willing to fill out the on-line questionnaire or who had access to computers, smartphones, or tablets were included. Even though selection bias could have affected the findings, our findings – with the exception of the higher mean scores for knowledge, which may reflect the overall demographic bias of the sample – are very similar to other studies with equivalent subgroups (13, 54–56). Data are self-reported and may have resulted in the participants inaccurately responding or giving socially desirable replies – a phenomenon that is prevalent in survey research (68). Another limitation of this sample was its biased educational level: nearly half had a Master’s or advanced degree, a characteristic that is not shared by the general public. Even though previous research has reported that men have little knowledge of CRC or screening exams regardless of education or ethnic group (58), our sample’s upward-biased educational level may have influenced their CRC and CRCS knowledge as well as the behavior of other variables in the model, including perceptions of MRN. Also, recruitment strategies inhibited random sampling and any attempts at generalizing to a larger population.

Another limitation relates to the survey’s focus on evaluating young adult AAM’s MRN, knowledge, perceptions, and attitudes regarding CRCS. The study was not designed to determine the influence of these men’s attitudes on their intention to screen for CRC. Although intention to carry out a behavior is a strong predictor of the behavior’s occurrence, unless the time lag in measuring both variables – intention and actual behavior – is short/small, the strength of the predictive relationship diminishes and becomes less useful for researchers (69). However, researchers who have access to measures of actual screening behaviors would do well, in the future, to explore the association between the variables examined in this study and young adult AAM’s intentions to screen *along with* actual screening behaviors (also outside the scope of this project). Furthermore, most of the measures we utilized in this study were originally designed in the context of different conceptual models or theories, and not the TPB. We incorporated these measures into a TPB framework, potentially introducing sizeable measurement error. One lesson this limitation highlights is that better measures are sorely needed for studying young adult AAM’s attitudes toward CRC and CRCS.

Other limitations this study suffered were the use of a convenience sample, and its small size, limiting the ability to generalize these findings to a larger population of AAM in the U.S. Nonetheless, the enthusiasm demonstrated by participants (some sent emails thanking for the opportunity to participate, and wishing the researcher success with the project) suggests this population might view participation in research positively if the researcher is perceived as trustworthy and the topic is relevant to the African American community (70, 71).

Finally, the decision to delete from the sample the surveys comprising incomplete data could have biased the analyses, given the data were not missing completely at random. Their numbers, however, were small enough that, the authors believe, their inclusion in the dataset would not have altered the findings significantly. Nonetheless, researchers who wish to pursue this topic in the future would do well to examine respondents’ discomfort with sexuality-related questions. Perhaps, because survey items were pre-tested with, mostly, graduate students, the authors were unable to capture the discomfort exhibited during subsequent data collection.

Despite these limitations, the study makes valuable contributions to understanding younger-than-50 adult AAM’s views of CRC and CRCS. As *the disease no one has to die from* (72), CRC is such a preventable and treatable condition when early detection occurs, that the gap which currently exists in the professional literature and in the research among young adult AAM should not be so extensive. Only four years ago, Powe, Faulkenberry, and Harmond noted that the number of intervention studies designed to increase CRCS among African Americans was relatively small (73). Findings from our study suggest that culturally relevant health promotion and early-intervention prevention programs for AAM should be developed addressing the salient factors shaping young AAM’s view of CRC and EDS behaviors. Furthermore, future research and intervention efforts should consider collaborating at the national, state, and community levels and utilizing family health history tools to change young adult AAM’s perceptions of barriers toward CRC, as well as their work status, and knowledge of their family history of cancer – three factors that – at least in this study’s sample – may shape these men’s future decisions to screen for CRC. Because this study was narrowly focused on a specific issue affecting a group of as-of-yet understudied African Americans, it provides a solid basis for developing structured health education interventions to increase young adult AAM’s intention to screen for CRC. As Simons-Morton, McLeroy, and Wendel propose,

“The more narrowly we can focus on a particular population group, the better we can assess the factors related to their health and behavior, and the better we can develop programs consistent with their needs” (p. 36) (64).

## Conflict of Interest Statement

The authors declare that the research was conducted in the absence of any commercial or financial relationships that could be construed as a potential conflict of interest.

## Footnotes

^1^The researchers ran two additional models, removing work status and health insurance, and the outcomes were no different than the one in Model 2.

^2^Additional analyses indicated the male role norms (MRN) variable could be construed as a single-factor variable (with a Cronbach’s alpha of 0.90), therefore, an additional model using the single-factor MRN variable was ran. Yet, findings remained the same.

## Abbreviations

AAM, African American men; ACS, American cancer society; CRC, colorectal cancer; CRCS, colorectal cancer screening; EDS, early detection screening; FOBT, fecal occult blood test; MRNI-SF, male role norms inventory-short form; TPB, theory of planned behavior.
